# Severe complications of tramadol overdose in Iran

**DOI:** 10.4178/epih.e2019026

**Published:** 2019-06-16

**Authors:** Paria Habibollahi, Alireza Garjani, Samad Shams Vahdati, Seyyed-Reza Sadat-Ebrahimi, Neda Parnianfard

**Affiliations:** 1 Pharmaceutical Analysis Research Center, Tabriz University of Medical Sciences, Tabriz, Iran; 2 Department of Pharmacology and Toxicology, Tabriz University of Medical Sciences, Tabriz, Iran; 3 Emergency Medicine Research Team, Tabriz University of Medical Sciences, Tabriz, Iran; 4 Research Center for Evidence-Based Medicine, Iranian Evidence-Based Medicine (EBM) Center: A Joanna Briggs Institute Affiliated Group, Health Management and Safety Promotion Research Institute, Tabriz University of Medical Sciences, Tabriz, Iran; 5 Drug Applied Research Center, Tabriz University of Medical Sciences, Tabriz, Iran

**Keywords:** Tramadol, Serotonin syndrome, Overdose, Complications, Iran

## Abstract

**OBJECTIVES:**

Severe complications of tramadol overdose have been reported; however, few large-scale studies have investigated this issue. Therefore, this study aimed to explore the presentation and complications of tramadol overdose in patients admitted to an intoxication referral center in northwestern Iran.

**METHODS:**

Patients with tramadol overdose admitted to Sina Teaching Hospital in Tabriz, Iran during 2013-2017 were included. For each patient, the following data were collected: demographics, previous drug or medication overdose, whether the patient was in the process of quitting drug use, ingested dose of tramadol and co-ingestants, Glasgow Coma Scale (GCS) score, clinical symptoms at the time of admission, and admission characteristics. Serotonin toxicity was diagnosed in patients who fit the Hunter criteria. Multiple logistic regression was performed to identify variables associated with the incidence of severe complications of tramadol overdose.

**RESULTS:**

In total, 512 cases of tramadol overdose were evaluated, of which 359 patients were included, with a median age of 41 years (range, 16-69) and a median tramadol dose of 1,500 mg (range, 500-4,000). The most frequent complications associated with tramadol overdose were hypertension (38.4%), tachycardia (24.8%), and seizure (14.5%). No serotonin toxicity was detected in patients. Having a GCS score <15, having taken a tramadol dose of >1,000 mg, being in the process of quitting drug use, being 30-49 years old, and male sex were significantly related to the incidence of severe complications of tramadol overdose.

**CONCLUSIONS:**

Although seizure was prevalent among Iranian patients with tramadol poisoning, serotonin toxicity and cardiogenic shock were rare findings.

## INTRODUCTION

Tramadol is one of the most widely used opioid drugs in medicine, and is mainly prescribed as a centrally-acting analgesic for patients suffering moderate to severe pain [1]. Tramadol is a synthetic 4-phenyl-piperidine analog of codeine, and its opioid effects are due to interactions with μ receptors, leading to effects on the noradrenergic and serotonergic systems through norepinephrine and serotonin reuptake inhibition, respectively, in the central nervous system (CNS) [2]. Moreover, it results in affecting the neurotransmitter gamma-aminobutyric acid (GABA) manifestations through increasing the levels of GABA mediators in the CNS [3]. Tramadol poisoning causes some mild to severe impairments in CNS, which manifest clinically as dizziness, nausea, vomiting, facial anesthesia, agitation, headache, ataxia, seizure, impaired consciousness, and coma [4]. Impaired consciousness can lead to hospitalization in roughly 10% of tramadol-poisoned patients, who are likely to have a longer stay in the intensive care unit [5]. Previous studies assumed that the manifestations of tramadol poisoning are mostly due to the inhibitory effects of tramadol on catecholamine and serotonin reuptake in the CNS [6,7]. It has been postulated in some case reports that tramadol is also associated with serotonin toxicity in the therapeutic range [8,9]. Serotonin toxicity involves a range of conditions, including neuromuscular excitement (e.g., hyperreflexia, clonus, and rigidity), autonomic effects (e.g., tremor, tachycardia, diaphoresis, flushing, and hyperthermia) and altered mental status (e.g., agitation, confusion, and anxiety) [10]. Moreover, cardiogenic shock has also been reported as a consequence of tramadol overdose [11]. A large number of patients with tramadol poisoning are annually admitted to the toxicology unit of Sina Teaching Hospital. Therefore, we investigated the initial presentation of patients with tramadol poisoning (coma, seizure, serotonin toxicity, etc.) admitted to the toxicology unit to shed further light on its possible complications, even rare complications.

## MATERIALS AND METHODS

In this cross-sectional study, all patients with tramadol overdose (i.e., doses greater than the maximum recommended dose of 400 mg) admitted to Sina Teaching Hospital in Tabriz, Iran from January 2013 to December 2017, were included. Using a pre-prepared checklist, data were gathered on patients’ demographics, previous history of overdose of any drugs or medications, whether they were in the process of quitting drug use, the Glasgow Coma Scale (GCS) score, ingested dose of tramadol and co-ingestants, clinical symptoms and noteworthy complications (e.g., coma, seizure, serotonin toxicity, cardiovascular events, respiratory depression, and mortality) during the time of admission, length of stay (LOS), the ward or unit of admission, and administration of mechanical ventilation. Data were gathered by trained staff members who were blinded to the results of the study. Tramadol ingestion and the ingested dose were confirmed through the patient’s medical history and the patient’s information was obtained from emergency medical services and the staff of the emergency department.

The Hunter criteria [12], which include the conditions of clonus (inducible, spontaneous, or ocular), agitation or diaphoresis, tremor, hyperreflexia, hypertonia, and temperature more than 38°C, were utilized to diagnose serotonin toxicity. Patients were excluded if there was uncertainty about tramadol ingestion and/or its dose, and if the dose of ingested tramadol was below 400 mg. Patients were also excluded if they had any previous convulsive disorder or had concomitantly ingested other drugs with tramadol.

Data analysis was performed using SPSS version 22 (IBM Co., Armonk, NY, USA). Data were reported as mean and standard deviation if they were normally distributed; otherwise, the median and range were reported. For normally distributed data, mean values were compared using the Student t-test; otherwise, they were analyzed using the Mann-Whitney U-test. To investigate the relationship of the study variables with the incidence of severe complications of tramadol overdose, through computing adjusted odds ratios (ORs) and their 95% confidence intervals (if the initial analysis yielded ORs of at least 1.12 for the relationship), multiple logistic regression was performed on data from 214 patients who experienced seizure, respiratory depression, or tachycardia following tramadol overdose. Backward elimination multiple logistic regression was performed to find the best predictors of the incidence of severe complications of tramadol overdose, including the GCS score, ingested tramadol dose, previous history of overdose with any drugs or medications, being in the process of quitting drug use, age, and sex. The p-values <0.05 were considered to indicate statistical significance.

### Ethics statement

This paper is a retrospective study so it did not need ethical consideration. Informed consent was waived by each patient included.

## RESULTS

In total, 512 cases of tramadol overdose were admitted to the toxicology unit during the study period, of which 153 cases were excluded, for the following reasons. In 16 patients, the dose was unclear, and in 25 patients, the dose was below 400 mg. Moreover, 94 cases had concomitantly ingested other drugs, including benzodiazepines, tricyclic antidepressants (TCAs), and other opiates. Eighteen patients had previous unprovoked seizures. The remaining 359 patients were included in the study and their data were meticulously recorded. The majority of patients (97.5%) were males. Only 15 (2.9%) patients had a previous history of overdose, while 162 (31.6%) were in the process of quitting drug use at the time of admission. The median age of patients was 41 years (range, 16-69). The median dose of tramadol was 1,500 mg (range, 500-4,000) (Table 1). A seizure occurred in 52 patients (14.5%), with evidence for generalized tonic-clonic seizures. In 80.8% of cases, the seizures occurred less than 24 hours after tramadol ingestion (Table 2).

The median dose in patients who experienced a seizure was 2,700 mg (range, 1,300-4,000), which was significantly higher than the median dose in patients without a seizure (p=0.001). Seizures also occurred in 7 of the 25 patients with an unclear dose. The time from ingestion to seizure was unknown for 12 patients, while the median time among the other 40 patients was 7.7 hours (range, 0.5-29.5).

Serotonin toxicity was not detected in any patients according to the Hunter criteria. In 108 (30.1%) patients, the GCS score was 15 or lower, and in 23 (6.4%) patients, it was less than 9. The median dose in patients with a GCS score of less than 9 was 3,100 mg (range, 2,850-3,750), which was significantly higher than that in patients with a GCS score of at least 9 (p=0.012). Ingestion of 1,100 to 4,000 mg of tramadol in 73 patients resulted in respiratory depression; the median dose in these patients was 2,750 mg (range, 2,350-4,000), which was also significantly higher than the median dose in patients without respiratory depression (p=0.034).

In total, 192 (53.5%) patients presented only nausea and/or vomiting, and 101 (28.1%) patients presented cardiovascular complications, of whom 89 (4.8%) had tachycardia, 138 (38.4%) had mild hypertension, and 19 (5.3%) had arrhythmia. However, no cases of cardiogenic shock occurred.

Seven patients were transferred to the intensive care unit (ICU) due to respiratory depression. The LOS in patients admitted to the toxicology ward was 28 hours (range, 12-136), while the patients who were transferred to the ICU had a significantly longer LOS (p=0.01). Three patients died, but their dose of tramadol was unknown. Naloxone was administered in 76 (21.2%) cases, with a median dose of 4.2 mg (range, 0.8-8.4). Naloxone helped 19 patients recover from respiratory depression and improved consciousness in 21 patients. However, no significant effect of naloxone was seen in the other 36 patients. No seizures occurred in patients who received naloxone.

Based on the results of the backward logistic regression model for variables associated with the incidence of severe complications of tramadol overdose, having a GCS score of 15 or lower (especially below 9), an ingested tramadol dose higher than 1,000 mg (especially above 2,000), being in the process of quitting drug use, being 30-49 years old (especially 40-49), and male sex were significantly related to the incidence of severe complications of tramadol overdose (all p<0.05) (Table 3).

## DISCUSSION

The most significant complications of tramadol overdose in our study were seizure, respiratory depression, and impaired consciousness. In accordance with the rarity of fatal consequences of tramadol overdose in the literature [13], we encountered a few cases of mortality, but the dose of tramadol in those cases was unknown. Despite the previous reports of serotonin toxicity in tramadol overdoses, no such cases were found in our study. This may have occurred because serotonin toxicity (diagnosed using the Hunter criteria) as a consequence of tramadol overdose is more likely to occur when tramadol is concomitantly ingested with other drugs, especially TCAs [14,15]; however, we excluded such patients from our study to analyze the effects of unadulterated tramadol.

Other common side effects, including nausea and vomiting, tachycardia, and increased blood pressure (systolic blood pressure >160 mmHg) occurred in the majority of patients, and were assumed to be due to the noradrenergic reuptake effects of tramadol [6]. Some of the symptoms of tramadol poisoning were similar to those reported previously for other synthetic opioids that have oral forms, including methadone and dextromethorphan. The gastrointestinal symptoms were comparable to those reported for methadone poisoning in a previous Iranian study [16]; however, respiratory depression was less common in tramadol poisoning. Hypotension was reported in methadone poisoning [17], but we did not encounter any casses of hypotension in our study of tramadol-poisoned patients. The prevalence of tachycardia in our patients was similar to that in dextromethorphan-poisoned patients reported in a large study in the USA; however, the rate of hypertension in tramadol-poisoned patients in the current study was higher than that in dextromethorphan-poisoned patients [14].

Previous studies in Iran on tramadol poisoning also reported that the majority of patients were male, with a similar age range to that of our study, including the studies of Taghaddosinejad et al. [18] (14-50 years), Goodarzi et al. [15] (17-45 years), and Shadnia et al. [16] (16-54 years). Regarding the dose of tramadol that induced seizures, previous studies in Iran have reported similar doses. Talaie et al. [19] reported that the mean dose of tramadol was 2,186.00±280.80 mg, and Taghaddosinejad et al. [18] reported a similar dose of 1,511±1,353 mg. However, Petramfar & Haghighi [20] reported a much lower dose than our study (363.2±303.1 mg) and Goodarzi et al. [15] reported a notably higher dose than our study (3,248±2,515 mg). This discrepancy may be due to ambiguity about the number of ingested tablets and their dose under the psychological stress of confronting a tonic-clonic seizure in the patients included in these studies. Additionally, some studies did not report a clear protocol for confirming the occurrence of seizure and the tramadol dose in those cases [15,20].

We observed that intentional overdose was the most common manner of tramadol poisoning. Young males were more common than females among cases of tramadol overdose, which is in agreement with other studies in Iran and other countries [6,16,19]. Being 30-49 years old (especially 40-49) was significantly related to the incidence of severe complications of tramadol overdose. People in their 40s and 50s experience more persistent pain than younger people due to the physical changes associated with aging. The high usage of tramadol among people aged 40-50 years may reflect their pervasive need to overcome physical pain [1,2].

In the present study, the time of seizures ranged from 30 minutes to about 30 hours post-ingestion; however, 80% of seizures occurred in the first 24 hours after ingestion, which is in agreement with studies conducted by Marquardt et al. [21], Jovanović-Cupić et al. [22], and Talaie et al. [19]. Single seizures were most frequent one, occurring in 84.3% of cases, while multiple seizures took place in 15.7% of cases, which is similar to other studies [23].

The results of our study demonstrated that seizures occurred in 14.5% of cases, which is similar to the findings of previous studies on tramadol overdose. A national pharmacovigilance study in France reported that seizures occurred in 6.7% of patients with tramadol poisoning [24]. Correspondingly, other investigations reported similar results, such as 8% in the study of Spiller et al. [6], 11% in the study of Ryan & Isbister [25], and 16% in the study of Maraquaradt et al. [21].

Naloxone was effective for 39 out of 76 patients in promoting recovery from respiratory depression or loss of consciousness. Although a seizurogenic effect of naloxone has been reported previously [26], we did not encounter such complications among our patients.

The wide variation in ingested tramadol doses that caused seizures in our study implies that seizures are unlikely to be dose-dependent. Consequently, it was not feasible to determine a lower limit for a tramadol dose that could result in a seizure. The study of Talaei et al. [19] also demonstrated that seizures were not dose-dependent. Although some studies postulated that seizures seemed to be dose-dependent, these studies included only a small number of tramadol overdose patients with seizures [18,22,23]. Therefore, it is conceivable that the patients who presented with seizures at lower doses in our study may have remained unnoticed in those studies. Future studies evaluating the complications of tramadol according to its serum concentration will yield more reliable information on this issue.

We acknowledge some limitations of our study. Although the researchers tried to report the most accurate ingested dose of tramadol, the possibility of imprecise claims remains a concern. Moreover, excluding patients with concurrent use of other drugs meant that some cases of tramadol overdose were not included.

In conclusion, we found that the severe complications of tramadol overdose included loss of consciousness, seizures, and respiratory depression. However, cardiogenic shock and serotonin toxicity were not observed in our study. Additionally, our results demonstrated that seizures due to tramadol were not a dose-dependent complication, as they occurred at a variety of doses. Naloxone was effective in most patients with severe complications and did not induce seizures in any of them. Additionally, several variables, including sex, age, GCS score, ingested tramadol dose, and being in the process of quitting drug use, were associated with the incidence of severe complications of tramadol overdose.

## Figures and Tables

**Table 1.** Patients’ demographics and clinical findings

Item	Value
Total (n)	359
Female	9
Male	350
Age (yr)	41 [16-69]
Dose (mg)	1,500 [500-4,000]
Seizures	52 (14.5)
Seizure tramadol dose (mg)	2,700 [1,300-4,000]
No seizure tramadol dose (mg)	1,450 [500-3,250]
Serotonin toxicity	0 (0.0)
Gastrointestinal symptoms	192 (53.5)
Intensive care unit	7 (1.9)
Respiratory depression	73 (18.5)
Intubated/ventilated	11 (3.6)
Length of stay (hr)	28 [12-136]
Max heart rate (bpm)	100 [88-140]
Max systolic BP (mmHg)	135 [100-160]

Values are presented as median [range] or number (%). Max, maximum; BP, blood pressure.

**Table 2.** Characteristics of seizures

Characteristics	n (%)
Generalized tonic-clonic seizures	52 (100)
Single	41 (78.8)
Multiple	11 (21.1)
Time of occurrence (after tramadol ingestion, hr)	
<24	42 (80.8)
≥24	10 (19.2)

**Table 3.** Associations between study variables and the incidence of severe complications of tramadol overdose based on a backward logistic regression model^1^

Variable	OR (95% CI)	p-value
GCS score		
<9	3.80 (2.00, 4.00)	<0.001
9-15	2.00 (0.15, 2.45)	0.024
>15	1.00 (reference)	
Tramadol dose (mg)		
>2,000	3.55 (2.37, 4.73)	<0.001
1,000-2,000	1.34 (0.02, 2.66)	0.039
<1,000	1.00 (reference)	-
In the process of quitting drug use		
Yes	2.99 (1.22, 3.75)	<0.001
No	1.00 (reference)	
Age (yr)		
30-39	2.23 (1.26, 3.38)	0.013
40-49	3.23 (1.17, 4.28)	<0.001
≥65	1.00 (reference)	
Sex		
Male	4.21 (2.09, 5.48)	<0.001
Female	1.00 (reference)	

GCS, Glasgow Coma Scale; OR, odds ratio; CI, confidence interval.

1 Logistic regression showed acceptable model fit (χ^2^(4)=4.463, p=0.043).
